# Effects of investor sentiment on stock volatility: new evidences from multi-source data in China’s green stock markets

**DOI:** 10.1186/s40854-022-00381-2

**Published:** 2022-08-23

**Authors:** Yang Gao, Chengjie Zhao, Bianxia Sun, Wandi Zhao

**Affiliations:** 1 School of Economics and Management, Beijing University of Technology, No. 100 Pingleyuan, Chaoyang District, Beijing, 100124 People’s Republic of China; 2 Department of Finance, Southern University of Science and Technology, Room 3#317, Wisdom Valley, No. 1088 Xueyuan Rd., Nanshan District, Shenzhen, 518055 People’s Republic of China; 3 School of Statistics, Capital University of Economics and Business, Fengtai District, Beijing, 100070 People’s Republic of China

**Keywords:** Internet sentiment, Trading sentiment, Realized volatility, Mediating effect, G11, G12, G14

## Abstract

The effect of investor sentiment on stock volatility is a highly attractive research question in both the academic field and the real financial industry. With the proposal of China's "dual carbon" target, green stocks have gradually become an essential branch of Chinese stock markets. Focusing on 106 stocks from the new energy, environmental protection, and carbon–neutral sectors, we construct two investor sentiment proxies using Internet text and stock trading data, respectively. The Internet sentiment is based on posts from Eastmoney Guba, and the trading sentiment comes from a variety of trading indicators. In addition, we divide the realized volatility into continuous and jump parts, and then investigate the effects of investor sentiment on different types of volatilities. Our empirical findings show that both sentiment indices impose significant positive impacts on realized, continuous, and jump volatilities, where trading sentiment is the main factor. We further explore the mediating effect of information asymmetry, measured by the volume-synchronized probability of informed trading (VPIN), on the path of investor sentiment affecting stock volatility. It is evidenced that investor sentiments are positively correlated with the VPIN, and they can affect volatilities through the VPIN. We then divide the total sample around the coronavirus disease 2019 (COVID-19) pandemic. The empirical results reveal that the market volatility after the COVID-19 pandemic is more susceptible to investor sentiments, especially to Internet sentiment. Our study is of great significance for maintaining the stability of green stock markets and reducing market volatility.

## Introduction

Along with the “carbon neutral and carbon peak” proposal by the Chinese government, green finance in China has recently attracted more attention. Green stocks in particular are favored by investors because of their essential social benefits. Unlike other stocks, green stocks denote common stocks with green attributes. The green attributes of these stocks are monitored by the China Securities Regulatory Commission and other relevant government departments. Specifically, green stocks are subject to strict environmental checks before listing, and they must satisfy environmental performance assessments and timely disclosure of environmental information after listing. Accordingly, investors may make investment decisions based on the disclosed environmental performance of the green stocks. Given the rich and open financing methods for green and environment-friendly enterprises, green stock markets can guide financial resources to the green industry, thereby providing enterprises with the impetus to reduce emissions. By 2020, the total market value of China’s green stock index exceeded 21 trillion CNY, becoming an essential branch of the whole stock market. That is, green stocks have become the investments of choice for many investors owing to their excellent long-term values. However, China’s green stock market is still in a rapidly emerging stage, and the majority of investors in China’s stock market are individual investors. Most individual investors exhibit irrational behaviors influenced by sentiment, thus affecting the stock market’s stability. Therefore, to monitor the movements of green stock market prices and develop green finance soundly, it is crucial to study the impact of investor sentiment on price volatility in China’s green stock markets.

Behavioral finance theory holds that investor sentiment plays an essential role in investment decisions, asset pricing, and risk management. In particular, investor sentiment has been theoretically verified to cause stock price movements, such as volatility or even jumps of the stock market in the short term. As a matter of fact, measurement of investor sentiment lays the foundation for subsequent application analysis. Because investor sentiment cannot be observed directly, the construction of sentiment indicators has always been a hot issue for scholars. Lee et al. (1991) first used the discount rate of closed-end funds as an investor sentiment proxy to explain the closed-end fund puzzle. Hereafter, investor sentiment measurement was formally proposed. Recent studies have divided investor sentiment indicators into three categories based on multi-source data. The first category includes subjective indicators produced through investigation, such as the American Association of Individual Investors (AAII) (He et al. 2019). Although such sentiment indicators can directly reflect investors’ psychological characteristics, investors may not consistently make transactions according to these sentiments.

The second type are objective indicators constructed from transaction data, such as mutual fund flows (Frazzini and Lamont 2008), which can indirectly reflect investor sentiment. However, a single indicator usually fails to fully reflect emotional changes, so a composite investor sentiment index combining various indicators was developed. Based on principal component analysis (PCA), Baker and Wurgler (2006) constructed the BW index, which measures market sentiment using six market trading indicators: closed-end fund discount rate, turnover rate, initial public offering (IPO) number, IPO first-day earnings, share ratio in newly issued bonds and stocks, and dividend premium. Since then, many scholars have further investigated and developed market sentiment indicators (Liang 2016; Hirshleifer et al. 2020). However, market sentiment indicators do not reflect investors’ sentiment toward a specific stock. With individual stock sentiment indicators, it is also easier to reveal the sensitivity of stock price fluctuations to investor sentiment. In addition, individual stock sentiment can be acquired based on daily frequency, while market sentiment is mostly monthly. Because investor sentiment is sensitive to information changes in the market, sentiment constructed based on daily data is more likely to capture the rapid changes in investor sentiment. Yang and Hu (2021) compared individual stock sentiment with market sentiment, verifying that the explanatory power of individual stock sentiment on individual stock returns is stronger than that of market sentiment. Therefore, our study constructs investor sentiments for individual stocks based on daily data.

In addition, with the development of the Internet and machine learning methods, the massive data sources resulting from investors’ interactions on the Internet have provided a third type of investor sentiment. Antweiler and Frank (2004) first acquired investors’ postings from Yahoo Finance and applied the naive Bayes method to classify text sentiment, developing a new way to construct Internet sentiment. Many scholars have employed machine learning methods to construct investor sentiment using text data from different online platforms. For instance, Li et al. (2020) utilized investors’ messages from Eastmoney Guba and quantified investor sentiment using the naive Bayes method. Furthermore, Duan et al. (2021) collected information related to the coronavirus disease of 2019 (COVID-19) from official news media and Sina Weibo and used support vector machines (SVM) to construct the COVID-19 sentiment. In addition to traditional machine learning approaches, deep learning methods, including convolutional neural networks (CNN), recurrent neural networks (RNN), and long short-term memory (LSTM), have also been applied to construct Internet sentiment (Jing et al. 2021; Carosia et al. 2021; Basiri et al. 2021). Recently, Google’s open-source project of bidirectional encoder representations from transformers (BERT) has offered new opportunities for natural language processing and has been successfully applied to a growing number of text classification problems (Leow et al. 2021; Carosia et al. 2021).

The efficient markets hypothesis posits that the deviation of financial asset prices from their fundamental value can be eliminated by arbitrageurs, while Shleifer and Summers (1990) point out that low information efficiency indicates limited arbitrage in the stock market. Stock market trades based on false subjective beliefs or information unrelated to the fundamentals of the company do occur. Kyle (1985) first proposed the concept of “noise trader,” and Black (1986) further defined noise traders as investors who cannot acquire inside information and irrationally regard unfiltered information as valid information to participate in transactions. Subsequently, based on the DSSW model, DeLong et al. (1990) revealed that non-fundamental signals from noise traders lead to an increase in the systemic risk of financial assets, indicating a relationship between sentiment and price volatility at the individual security level. The more irrational arbitrageurs trade on noisy signals, the greater the price swings. Baker and Wurgler (2007) further pointed out that the impact of investor sentiment on stock prices is related to the characteristics of companies. Specifically, companies that are young, unprofitable, highly volatile, distressed, and seeking growth, as well as companies that have small market capitalization and non-dividend-paying stocks, are generally affected by sentiment. Since then, many scholars have analyzed the impact of investor sentiment on stock price volatility and found that investor sentiment can significantly exacerbate stock volatility (Siganos et al. 2017; Rupande et al. 2019; Jiang and Jin 2021). However, the existing studies only consider the single effect of trading sentiment or Internet sentiment on stock volatility. Few studies in the literature have simultaneously examined the influence of the two sentiment proxies on volatility. Our study combines multi-source heterogeneous data to construct both the Internet sentiment and trading sentiment of individual stocks. We then simultaneously examine the impacts of the two investor sentiments on Chinese green stocks’ volatilities. In particular, volatility is decomposed into continuous volatility and jump volatility, and the differences in the influences of the two sentiments on continuous volatility and jump volatility are investigated further.

Market participants tend to believe that homogeneous information is not evenly distributed in the market (Javakhadze et al. 2014). Affected by investors’ ability to seek information and the degree of information disclosure, information asymmetry is expected in the stock market. Kyle (1985) and Easley and O’hara (1987) found that informed traders will take advantage of their information to profit from uninformed investors with optimal trades. Trading frequency increases when investor sentiment is relatively high, improving the liquidity level, which is important for informed investors’ transactions, and reducing transaction costs. Earlier studies have also evidenced the impact of investor sentiment on information asymmetry (Li et al. 2022). Further, Jindra and Moeller (2020) pointed out that the uncertainty of company valuation comes from information asymmetry. Information asymmetry, usually reflected by an adverse selection cost such as bid-ask spread, could prompt stock market volatility and play an essential role in stock price fluctuations. Easley et al. (1996) first defined the probability of informed trading (PIN) as a measure of information asymmetry. However, the PIN often encounters an overflow problem in the calculation process. To solve this calculation problem, Easley et al. (2011) further introduced the volume-synchronized probability of informed trading (VPIN). The existing literature reveals that the VPIN can cause an imbalance in intraday orders (Wei et al. 2013), resulting in short-term volatility (Wei et al. 2013; Bjursell et al. 2017). In a market with asymmetric information, the greater the proportion of informed traders who execute trades based on private information, the larger the impacts on market volatility (Li and Wen 2019). Concerning the interdependence between investor sentiment, information asymmetry, and price volatility, few studies have investigated the mediating role of information asymmetry in the effect of investor sentiment on volatilities. Therefore, we select the VPIN, a widely used metric for measuring information asymmetry, as the mediating variable and discuss how investor sentiment affects volatility by changing the VPIN.

Moreover, the existing literature has verified that investor sentiment can significantly cause stock market volatility. Baker and Wurgler (2007) revealed that investor sentiment even imposes more severe impacts on the stock market than fundamentals in uncertain periods. Because of the outbreak of COVID-19, worldwide stock markets have been facing severe challenges. Recent studies have explored the changes in investor sentiment and their impact on the stock market. For example, Pagano et al. (2021) revealed that Robinhood retail investors responded quickly to overnight returns, pursuing both momentum and contrarian strategies. In addition, Smales (2021) pointed out that investors paid more attention to the coronavirus during the COVID-19 crisis, and investor attention is positively correlated with stock market volatility. Sun et al. (2021) also found heterogeneity in the impact of coronavirus-related news (CRNs) and economic-related announcements (ERAs) associated with the COVID-19 outbreak on investment sentiment in different countries. Moreover, Huynh et al. (2021) used a series of coronavirus-related sentiment indices, including media coverage, fake news, panic, sentiment, media hype, and infodemics, to construct the feverish sentiment index at the national level. They found that investor sentiments in 17 countries showed a strong correlation, and the feverish sentiment index can positively predict the stock volatility of several countries. Recently, Anastasiou et al. (2022) constructed a novel positive search volume index for COVID-19 (COVID19 +) and found that the rise of COVID-19 + could reduce investors’ crisis sentiment and ease stock market volatility. Therefore, because of the COVID-19 outbreak, our study divides the sample into pre-and post-pandemic subsamples and examines whether there are any differences in the impacts of the two investor sentiment proxies on volatilities in different periods.

Our study contributes to the literature in two ways. The existing research usually considers either trading or Internet sentiment when exploring the impact of investor sentiment on stock volatility, and few studies analyze the role of VPIN between investor sentiment and volatility. Therefore, we first construct both Internet and trading sentiments based on multi-source data and then analyze their impacts on the price movements of China’s green stocks. We find the two sentiments are positively correlated with the VPIN, and confirm the mediating role of the VPIN in the effects of investor sentiments on stock price volatilities. Second, considering that the existing literature rarely compares the similarities and differences of the impact of investor sentiment on realized, continuous, and jump volatility, we decompose realized volatility into continuous volatility and jump volatility and analyze the differences in the influence of investor sentiment on volatilities. Moreover, we conduct further analysis by dividing the sample into different stock boards and different periods. We find that the impacts of Internet sentiment on jump volatility for the small and medium enterprise (SME) and growth enterprise market (GEM) boards seem relatively limited. Moreover, by dividing the sample into two period, before and after the COVID-19 pandemic, we find that investor sentiments have more pronounced effects on stock volatilities after the pandemic, especially for Internet sentiment. However, the mediating effect of the VPIN in the impact of trading sentiment on volatility after the pandemic is more prominent than before the pandemic.

The remainder of this paper is organized as follows. Section 2 describes the theoretical analysis and research hypotheses. Section 3 presents the research design of our studies. Section 4 conducts the empirical analysis. Section 5 presents further analyses based on the subsamples before and after the pandemic. Finally, Sect. 6 provides a brief conclusion.

## Theoretical analysis and research hypothesis

The Green stock market plays a vital role in encouraging listed companies to disclose environmental information, guiding social capital to enter the field of environmental protection. However, the economic benefits of green stocks are mainly reflected in the long run. The emerging Chinese green stock market is still rapidly developing, and external supervision has not yet been perfected. This may lead green stocks to have insufficient short-term operating performance, which would be reflected in the price volatility in the short term. In addition, the price volatility of the stock market is mainly determined by the supply–demand relationship. When the buyers’ power is greater than that of the sellers’, the stock market demand is greater than the supply. This will cause a rise in the stock price, and vice versa. Behavioral finance holds that investors’ investment psychology will affect stock price fluctuations. For example, Barberis et al. (1998) revealed that investors may be affected by representational bias when dealing with new information, which is manifested as overemphasizing recent information but ignoring historical aggregate data. When investors pay too much attention to short-term good news, they will overestimate future stock prices; and once future earnings fail to meet expectations, investors will get panicked and stock prices will then fall. Moreover, Barber and Odean (2008) developed a price pressure hypothesis to explain the impact of investor sentiment on stock prices. The theory holds that investors, because of their limited time and energy, usually only invest stocks that attract their attention. An increase in investor attention will put upward pressure on stocks in the short term, and then reverse.

In theory, the price fluctuations of financial assets usually display a leverage effect. That is to say, bad news tends to induce higher volatility than good news does. Especially after the COVID-19 outbreak, the usual information disclosure may fail to satisfy investors’ thirst, and the impact of information on the stock market will be more powerful. The existing literature also reveals a significant correlation between investor sentiment and volatility. For example, Rupande et al. (2019) pointed out that irrational investor sentiment exacerbates stock return volatility, and they proposed that investor sentiment is a risk factor in asset pricing. Audrino et al. (2020) applied text data to construct investor sentiment and revealed that the accuracy of volatility prediction is significantly improved with the inclusion of investor sentiment. Abdelmalek (2021) also confirmed that a rise in investor sentiment would increase the volatility and instability of the stock market. Thus, we propose the first hypothesis as follows:

### H_1_

High investor sentiment exacerbates the return volatility of green stocks.

Because of differences in investors’ access to information and their ability to process information, the asynchronous transmission of information in the stock market results in information asymmetry. Daniel et al. (1998) revealed that public information and private information in the market exert asymmetric effects on investors. Some investors will overestimate the accuracy of signals sent by private information, and overconfidence will cause private signals to have higher weights than prior information, causing an excessive stock-price reaction. If individual investors exhibit stronger behavioral biases in hard-to-value stocks, relatively informed investors may exploit these biases for gains. Kumar (2009) applied the consumer sentiment index and found that individual investors exhibit more substantial behavioral bias when stocks are challenging to value and market uncertainty reaches a high level. Therefore, investors with an information advantage tend to take advantage of these deviations to yield returns, and thus have a higher probability of informed trading. The reasons for this phenomenon may lie in two aspects. On the one hand, higher investor sentiment indicates more active trading activity, which is beneficial for informed traders to hide their trading activities, thus aggravating the level of information asymmetry (Zhu et al. 2017). On the other hand, the rise of Internet social media has led to the disclosure of vast quantities of stock-related information, and the role of social media has become more complicated. Some managers of public firms may conceal bad news in consideration of their short-term interests, and many speculators will not readily share inside information on social platforms because of the cost they incurred to acquire the inside information. However, when investor sentiment turns high, investors tend to overreact to the information they obtain, leading to a herd effect. This increase in trading activity will reduce the transaction cost of informed traders, thereby increasing the proportion of informed traders’ transactions. Using the VPIN to measure the degree of information asymmetry, our second hypothesis is therefore proposed:

### H_2_

High investor sentiment is positively correlated with the VPIN.

Information theory in finance holds that the trades with informed traders will damage the interests of uninformed traders, and the order imbalance caused by information trading exacerbates stock price volatility. Informed traders use information from outside the market to seek arbitrage opportunities, which interferes with the investment direction of other investors. When informed traders conduct transactions, the external information they possess will be reflected in the stock price, thereby causing stock price volatility. The more frequently informed traders trade, the more volatile the stock prices become. The existing literature has verified the impact of information asymmetry on stock market volatility. For instance, Low et al. (2018) found that an increase in VPIN can effectively predict high volatility in several stock indices. Yildiz et al. (2020) found a positive correlation between return volatility and VPIN. This finding is expected because information consolidation is positively correlated with return volatility (Barclay et al. 1990; French and Roll 1986), and VPIN is designed to capture large amounts of information. Yang and Xue (2021) improved the VPIN model based on neural networks and high-frequency data, and confirmed that the VPIN is a good signal for information trading and price volatility. According to H_2_, high investor sentiment may intensify the degree of information asymmetry. Thus, we propose the third hypothesis of our study.

### H_3_

Investor sentiment affects stock return volatility through the mediating role of the VPIN.

## Research design

### The sample

The green stock index is generally used to evaluate stocks with green attributes. Specifically, China’s green stock index can be roughly divided into the sustainable development, environmental protection industry, new energy, and green environment sectors. To investigate the influence of investor sentiment on the volatility of the green stock market, we select 106 stocks from the new energy, environmental, and carbon–neutral sectors listed in China’s stock markets. Details about these stocks are shown in Table A1 of the Appendix. All of the selected stocks are above grade B, according to the environment, society, and government (ESG) ratings in the Wind database. The ESG score of these stocks reaches 6.3266, on average. In contrast, the average ESG score of all stocks in China’s A-share market is 5.9376, indicating that the selected stocks do have higher ESG scores on the whole. The sample interval ranges from June 3, 2019 to December 31, 2020, and the frequency of all variables is daily. We select Eastmoney Guba (https://guba.eastmoney.com/) as the text data source for Internet sentiment. We use Python to write the crawler program and crawl all titles relating to each sample stock from June 3, 2019 to December 31, 2020. The stock code, number of readings and comments, author, and post time of each title are also obtained. We then delete closed and meaningless titles, such as forwards and pictures. Finally, a total of 2,608,027 titles are ready for use. In the following, FinBERT will be used for text sentiment classification to convert text into structured data and further calculate the daily Internet sentiment. In addition, we download daily trading indicators from the Wind and CSMAR databases as proxy variables to construct daily trading sentiment. Notably, the daily realized volatility and its decompositions are constructed based on 5-min high-frequency data, and the intraday data comes from the RESSET database. The VPIN and control variable data also come from the Wind and CSMAR databases.

**Table 1:** Variable definitions

	Variable	Meaning
Explained variables	*RV*_*i,t*_	The realized volatility of stock *I* on day *t*
	*RBV*_*i,t*_	The continuous volatility of stock *I* on day *t*
	*Jump*_*i,t*_	The jump volatility of stock *I* on day *t*
Explanatory variables	*SentiIntern*_*i,t*_	The Internet sentiment of stock *I* on day *t*
	*SentiTrade*_*i,t*_	The trading sentiment of stock *I* on day *t*
Mediating variable	*VPIN*_*i,t*_	The probability of informed trading of stock *I* on day *t*
Control variables	*Return*_*i,t*_	The return of stock *I* on day *t*
	*BM*_*i,t*_	The book-to-market ratio of stock *I* on day *t*
	*Size*_*i,t*_	The market value of stock *I* on day *t*
	*SenNum*_*i,t*_	The number of posts of stock *I* on day *t*
	*CS*_*t*_	The credit spread of the market on day *t*
	*TS*_*t*_	The term spread of the market on day *t*
	*Tues*_*t*_*/Wed*_*t*_*/Thur*_*t*_*/Fri*_*t*_	The four weekday-effect dummies on day *t*

### Variable constructions

#### Investor sentiment

*Internet sentiment.* The BERT method is a deep interactive pre-trained language model based on the semantic understanding derived from the transformer. The BERT uses transformer encoders as feature extraction tools and adds position encoding to recognize position information to understand language order. In addition, it uses self-attention to improve the computing capability of the model and adopts the scaled dot product as the attention scoring function. The output vector sequence can be written as

1 $$Attention(Q,K,V) = softmax\left( {\frac{{Q^{T} K}}{{\sqrt {d_{k} } }}} \right)V,$$

where *Q* represents the query vector, *K* denotes the key vector, *V* is the value vector, $1/\sqrt {d_{k} }$ is the scaling factor, and *softmax* is the normalization function. Furthermore, BERT introduces a multi-head self-attention mechanism to extract more interactive information in multiple spaces. The results of the attention function calculation are then processed by layer normalization, which is defined as follows:

2 $$LN(x_{i} ) = \alpha \times \frac{{x_{i} - \mu_{L} }}{{\sqrt {\sigma_{L}^{2} + \varepsilon } }} + \beta ,$$

where $\mu_{L}$ denotes the mean value of net input $x_{i}$ of neurons in layer *L*, $\sigma_{L}^{2}$ is the variance of net input $x_{i}$ of neurons at layer *L*, and $\alpha$ and $\beta$ represent the parameter vectors of scaling and translation, respectively. In addition,$\varepsilon$ is an extremely small constant set for numerical stability. After normalization, feed-forward neural networks composed of two full connections are used for the relevant learning. The BERT uses the above basic mechanism to yield a pre-trained language model through unsupervised training with massive text.

**Fig. 1:**
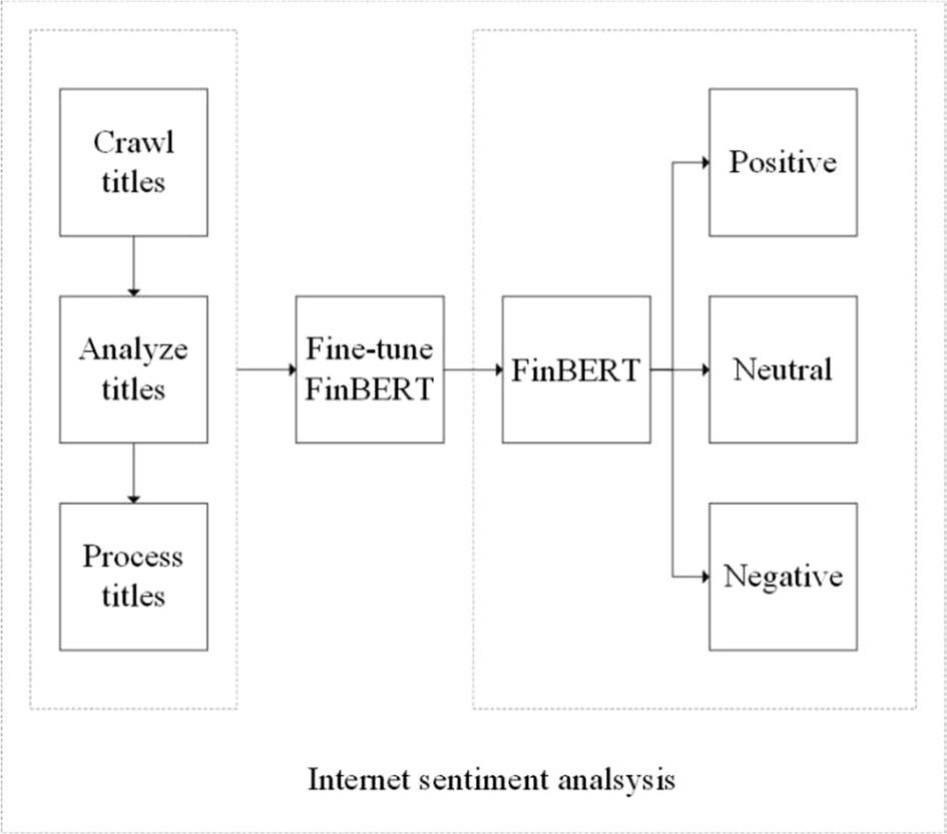
Process of sentiment classification

Although the BERT is a milestone in processing the sentiment classification of Chinese text, its application in the financial field still needs to be improved. Therefore, Entropy Jane Technology trained the FinBERT pre-training language model based on BERT, using one million financial and economic news articles, nearly two million various research papers, company announcements, and about one million financial encyclopedia entries in 2020. We add a specific task output layer and selected 30,000 titles from the Eastmoney Guba training output layer for application to the target task. The classifier labels negative sentiment as − 1, neutral sentiment as 0, and positive sentiment as 1. The overall process is illustrated in Fig. 1.

Then, referring to Antweiler and Frank (2004), we construct the Internet sentiment in Eq. (3).

3 $$SentiIntern_{i,t} = ln\left[ {\left( {1 + M_{pos,i,t} } \right)/\left( {1 + M_{neg,i,t} } \right)} \right],$$

where $SentiIntern_{i,t}$ represents the Internet investor sentiment of stock* I* on day *t*, $M_{pos,i,t}$ indicates the number of positive titles of stock* I* on day *t*, and $M_{neg,i,t}$ represents the corresponding number of negative titles.

*Trading sentiment*. To measure investor sentiment systematically and comprehensively, we select several investor sentiment proxies to synthesize the trading sentiment from multiple indicators. Following Fu et al. (2021), we employ the principal component analysis (PCA) method to construct a firm-specific trading sentiment based on three underlying indicators, including turnover rate (*TURN*), buy-sell imbalance (*BSI*), and price-earnings ratio (*PE*).

The *TURN* indicator is calculated as the share-trading volume divided by the number of outstanding shares. Baker and Wurgler (2006) believe that the turnover rate can measure the investor sentiment and reflect the active degree of market transactions. Generally speaking, a high turnover rate indicates high demand from emotional investors, which can easily cause stock price instability (Han and Li 2017).

The *BSI* indicator is constructed by the imbalance between active buying and selling amounts. Kumar and Lee (2006) first include *BSI* in the construction of retail sentiment. Since then, *BSI* has been widely used to construct investor sentiment (Gao and Liu 2020; Li 2021). The calculation of *BSI* is

4 $$BSI_{i,t} = \frac{{BV_{i,t} - SV_{i,t} }}{{BV_{i,t} + SV_{i,t} }},$$

where *BV*_*i,t*_ is the amount of active buying of stock *I* in period *t*, and *SV*_*i,t*_ denotes the active selling orders of stock *I* in period *t*. Specifically, a positive *BSI* indicates that investors are in a high mood, and a negative *BSI* means that investors are depressed.

*PE* represents the ratio of a stock’s price divided by the earnings per share. The high PE ratio partly reflects investors’ recognition of a company’s growth potential. Suppose a stock’s PE ratio is much higher than its peers’. In this case, it is generally believed that the company’s future earnings will proliferate, and investor sentiment is relatively high. As the core and most commonly used measure of enterprise valuation, the PE ratio is widely used in the construction of trading sentiment (Cheema et al. 2020).

In consideration of the contemporaneous or lag interdependence between these three underlying proxies and investor sentiments, we first produce the lag-one terms of the sentiment indicators. We then conduct the PCA to develop a composite index of firm-specific investor sentiments based on the six indicators, including both the contemporaneous and lag-one terms of the three underlying proxies. The correlation comparison analysis reveals that the contemporaneous terms of *TURN, PE,* and the lag-one term of *BSI* take the first three places. Thus, we apply the PCA method on these three proxies and construct the firm-specific sentiment by retaining the first two principal components, whose cumulative variance contribution rate reaches 73%, as shown in Eq. (5).

5 $$\mathop {SentiTrade}\nolimits_{i,t} = 0.365TURN_{i,t} + 0.259BSI_{i,t - 1} + 0.551PE_{i,t}$$

#### Volatility and its decompositions

To measure daily volatility, we adopt the realized volatility (*RV*) proposed by Andersen and Bollerslev (1998), which is based on 5-min high-frequency data. Given stock *I* with *n* intraday returns on trading day *t*, the realized volatility is then defined as the square of the 5-min intraday returns, and the specific formula is

6 $$RV_{i,t} = \sum\limits_{j = 1}^{n} {r_{i,t(j)}^{2} } ,$$

where *r*_*i,t(j)*_ is the logarithmic return of the *j*-th 5-min interval of stock *I* on day *t*, *j* = 1,2,…,*n*. *RV* can be considered as a consistent estimate of the true volatility under a continuous diffusion process assumption of stock prices. However, the continuous-time financial theory posits that the asset price without arbitrage is a semi-martingale process. That is, the price process is not necessarily continuous and may contain jumps. Therefore, Barndorff-Nielsen and Shephard (2004, 2006) proposed a non-parametric estimation method called the realized bi-power variation (*RBV*) to filter jump volatility, as shown in Eq. (7).

7 $$RBV_{i,t} = \mathop \mu \nolimits_{1}^{ - 2} \mathop {\left( {1 - 2n^{ - 1} } \right)}\nolimits^{ - 1} \sum\limits_{j = 3}^{n} {\left| {r_{i,t(j)} } \right|} \left| {r_{i,t(j - 2)} } \right|,$$

where $\mu_{1}$ is a constant equal to $\left( {2/\pi } \right)^{1/2}$. Assuming that the logarithmic price process is a semi-martingale and finite jump process, the *RBV* converges to the integral variance in probability. Then, the difference between the realized volatility and the realized bi-power variation is indeed a consistent estimate of the jump volatility. In theory, the value of the jump volatility should be positive, but there may be an empirical case where *RV*_*i,t*_ is less than *RBV*_*i,t*_. Therefore, based on the method of Andersen et al. (2007), we define *Jump*_*i,t*_ as

8 $$Jump_{i,t} = max\left\{ {RV_{i,t} - RBV_{i,t} ,0} \right\}.$$

#### Information asymmetry and control variables

*Information asymmetry* The probability of informed trading (PIN) refers to the probability that a transaction comes from an informed trader with private information, and it always performs as an essential indicator in measuring the degree of information asymmetry. The higher the PIN, the more severe the degree of information asymmetry. Because overflow problems are often encountered in the calculation of the PIN, Easley et al. (2011) developed a VPIN estimator to solve this problem. The VPIN method divides the total transaction volume of a trading day into *n* transaction buckets with equal volumes, and the transaction volume of each transaction bucket is denoted as *V*. Informed traders will choose the direction of buying or selling based on their private information, resulting in an imbalance in buying or selling transactions. In calculating the imbalance of each transaction bucket, a transaction is regarded as a buyer’s order if the trading amount of the present transaction is higher than the previous transaction. Otherwise, the transaction is denoted as a seller’s order. Referring to Easley et al. (2012), the series of price differences between adjacent transactions in each bucket is standardized and incorporated into the standard normal distribution function. We can then compute the active buying or selling volume of each transaction. Specifically, the VPIN can be computed by Eq. (9).

9 $$VPIN = \frac{{\sum\nolimits_{\tau = 1}^{n} {\left| {V_{\tau }^{B} - V_{\tau }^{S} } \right|} }}{nV}.$$

Here, *n* denotes the number of buckets, usually taken as 50. $V_{\tau }^{B}$ represents the active buying volume of each transaction, and $V_{\tau }^{S}$ is the active selling volume of each transaction.

*Control variables.* Following Antweiler and Frank (2004) and Sabherwal et al. (2011), we employ stock returns (*Return*), firm size (*Size*), book-to-market ratio (*BM*), and the number of posts (*SenNum*) as the control variables. Moreover, referring to John and Li (2021), we further add the market credit spread and term spread as control variables. The credit spread adopts the interest rate difference between the China Securities Index (CSI) corporate bond AA + and the government bond with a maturity of one year. The term spread is the interest difference between the 10-year and 1-year government bonds. Early studies reveal that stock market volatility is closely related to the weekday or calendar effect (Doyle and Chen 2009; Keef et al. 2009). We therefore add the weekday effect and introduce the following four dummy variables, *Tues*_*t*_, *Wed*_*t*_, *Thur*_*t*_, and *Fri*_*t*_, into the regression models.

$$Tues_{t} = \left\{ {\begin{array}{*{20}l} {1,} \hfill & {{\text{if}}\;t\;{\text{is}}\;{\text{Tuesday}}} \hfill \\ {0,} \hfill & {\text{others,}} \hfill \\ \end{array} } \right.\;Wed_{t} = \left\{ {\begin{array}{*{20}l} {1,} \hfill & {{\text{if}}\;t\;{\text{is}}\;{\text{Wednesday}}} \hfill \\ {0,} \hfill & {\text{others,}} \hfill \\ \end{array} } \right.\;Thur_{t} = \left\{ {\begin{array}{*{20}l} {1,} \hfill & {{\text{if}}\;t\;{\text{is}}\;{\text{Thursday}}} \hfill \\ {0,} \hfill & {\text{others,}} \hfill \\ \end{array} } \right.\;Fri_{t} = \left\{ {\begin{array}{*{20}l} {1,} \hfill & {{\text{if}}\;t\;{\text{is}}\;{\text{Friday}}} \hfill \\ {0,} \hfill & {{\text{others}}} \hfill \\ \end{array} } \right..$$

Detailed variable definitions are given in Table 1.

### Model construction

#### Baseline model

To investigate the impact of investor sentiment on the realized volatility of green stocks, we first include the trading sentiment to conduct a preliminary study employing the following regression:

10 $$\begin{aligned} RV_{i,t} = & \alpha_{11} + \beta_{11} \mathop {SentiTrade}\nolimits_{{i,t{ - }1}} + \sum\limits_{m = 1}^{p} {\gamma_{m1} } Controls_{{i,t{ - }1}} + \lambda_{11} RV_{i,t - 1} \\ & \quad + \alpha_{i} + \phi_{11} Tues_{t} + \phi_{12} Wed_{t} + \phi_{13} Thur_{t} + \phi_{14} Fri_{t} + \varepsilon_{1,i,t} . \\ \end{aligned}$$

Specifically, we adopt the lag-one terms of the independent variables in all regressions to avoid endogeneity. Considering the continuity of price fluctuation, we add the lag-one terms of the dependent variable as a control variable. The Internet sentiment is then added to examine its effect on realized volatility, as shown in Eq. (11).

11 $$\begin{aligned} RV_{i,t} = & \alpha_{12} + \beta_{12} \mathop {SentiTrade}\nolimits_{{i,t{ - }1}} + \delta_{1} \mathop {SentiIntern}\nolimits_{{i,t{ - }1}} + \sum\limits_{m = 1}^{p} {\gamma_{m2} } Controls_{{i,t{ - }1}} + \lambda_{12} RV_{i,t - 1} \\ & \quad + \alpha_{i} + \phi_{21} Tues_{t} + \phi_{22} Wed_{t} + \phi_{23} Thur_{t} + \phi_{24} Fri_{t} + \varepsilon_{2,i,t} . \\ \end{aligned}$$

Under the assumption of a discontinuous diffusion process of stock prices, the realized volatility can be decomposed into continuous and jump volatilities. To further investigate whether the impact of investor sentiment on volatility is mainly attributable to continuous or jump volatility, we replace the realized volatility with continuous volatility in Eqs. (10) and (11). The specific equations are as follows:

12 $$\begin{aligned} RBV_{i,t} = & \alpha_{21} + \beta_{21} \mathop {SentiTrade}\nolimits_{{i,t{ - }1}} + \sum\limits_{k = 1}^{p} {\gamma_{k1} } Controls_{{i,t{ - }1}} + \lambda_{21} RBV_{i,t - 1} \\ & \quad + \alpha_{i} + \phi_{31} Tues_{t} + \phi_{32} Wed_{t} + \phi_{33} Thur_{t} + \phi_{34} Fri_{t} + \varepsilon_{3,i,t} , \\ \end{aligned}$$

13 $$\begin{aligned} RBV_{i,t} = & \alpha_{22} + \beta_{22} \mathop {SentiTrade}\nolimits_{{i,t{ - }1}} + \delta_{2} \mathop {SentiIntern}\nolimits_{{i,t{ - }1}} + \sum\limits_{k = 1}^{p} {\gamma_{k2} } Controls_{{i,t{ - }1}} + \lambda_{22} RBV_{i,t - 1} \\ & \quad + \alpha_{i} + \phi_{41} Tues_{t} + \phi_{42} Wed_{t} + \phi_{43} Thur_{t} + \phi_{44} Fri_{t} + \varepsilon_{4,i,t} . \\ \end{aligned}$$

Similarly, we examine the influence of investor sentiment on jump volatility, as shown in Eqs. (14) and (15).

14 $$\begin{aligned} Jump_{i,t} = & \alpha_{31} + \beta_{31} \mathop {SentiTrade}\nolimits_{{i,t{ - }1}} + \sum\limits_{k = 1}^{p} {\gamma_{k1} } Controls_{{i,t{ - }1}} + \lambda_{31} Jump_{i,t - 1} \\ & \quad + \alpha_{i} + \phi_{51} Tues_{t} + \phi_{52} Wed_{t} + \phi_{53} Thur_{t} + \phi_{54} Fri_{t} + \varepsilon_{5,i,t} , \\ \end{aligned}$$

15 $$\begin{aligned} Jump_{i,t} = & \alpha_{32} + \beta_{32} \mathop {SentiTrade}\nolimits_{{i,t{ - }1}} + \delta_{3} \mathop {SentiIntern}\nolimits_{{i,t{ - }1}} + \sum\limits_{k = 1}^{p} {\gamma_{k2} } Controls_{{i,t{ - }1}} + \lambda_{32} Jump_{i,t - 1} \\ & \quad + \alpha_{i} + \phi_{61} Tues_{t} + \phi_{62} Wed_{t} + \phi_{63} Thur_{t} + \phi_{64} Fri_{t} + \varepsilon_{6,i,t} . \\ \end{aligned}$$

#### Mediating effect model

We further verify the mediating effect of the VPIN in the influence of investor sentiment on stock volatilities. Specifically, based on Eq. (10), we construct the mediating effect model to examine the specific path of investor sentiment on volatility, as shown in Eqs. (16) and (17).

16 $$\begin{aligned} VPIN_{i,t} = & \omega_{11} + \xi_{11} \mathop {SentiTrade}\nolimits_{{i,t{ - 1}}} + \sum\limits_{{u{ = }1}}^{p} {\gamma_{u1} } Controls_{{i,t{ - }1}} + \varphi_{11} VPIN_{i,t - 1} \\ & \quad + \omega_{i} + \psi_{11} Tues_{t} + \psi_{12} Wed_{t} + \psi_{13} Thur_{t} + \psi_{14} Fri_{t} + \varepsilon_{7,i,t} , \\ \end{aligned}$$

17 $$\begin{aligned} RV_{i,t} = & \alpha_{14} + \beta_{14} \mathop {SentiTrade}\nolimits_{{i,t{ - }1}} + \theta_{1} \mathop {VPIN}\nolimits_{{i,t{ - }1}} + \sum\limits_{{\text{w = 1}}}^{p} {\gamma_{w4} } Controls_{{i,t{ - }1}} + \lambda_{14} RV_{i,t - 1} \\ & \quad + \alpha_{i} + \phi_{71} Tues_{t} + \phi_{72} Wed_{t} + \phi_{73} Thur_{t} + \phi_{74} Fri_{t} + \varepsilon_{8,i,t} . \\ \end{aligned}$$

In addition, our study also investigates the impact of the VPIN on volatility with the simultaneous existence of both Internet and trading sentiments. That is, we include the Internet sentiment into Eqs. (16) and (17), as shown in Eqs. (18) and (19).

18 $$\begin{aligned} VPIN_{i,t} = & \omega_{12} + \xi_{12} \mathop {SentiTrade}\nolimits_{{i,t{ - 1}}} + \delta_{4} \mathop {SentiIntern}\nolimits_{{i,t{ - 1}}} { + }\sum\limits_{{u{ = }1}}^{p} {\gamma_{u2} } Controls_{{i,t{ - }1}} + \varphi_{12} VPIN_{i,t - 1} \\ & \quad + \omega_{i} + \psi_{21} Tues_{t} + \psi_{22} Wed_{t} + \psi_{23} Thur_{t} + \psi_{24} Fri_{t} + \varepsilon_{9,i,t} , \\ \end{aligned}$$

19 $$\begin{aligned} RV_{i,t} = & \alpha_{15} + \beta_{15} \mathop {SentiTrade}\nolimits_{{i,t{ - }1}} + \delta_{5} \mathop {SentiIntern}\nolimits_{{i,t{ - }1}} { + }\theta_{2} \mathop {VPIN}\nolimits_{{i,t{ - }1}} + \sum\limits_{{\text{w = 1}}}^{p} {\gamma_{w5} } Controls_{{i,t{ - }1}} + \lambda_{15} RV_{i,t - 1} \\ & \quad + \alpha_{i} + \phi_{81} Tues_{t} + \phi_{82} Wed_{t} + \phi_{83} Thur_{t} + \phi_{84} Fri_{t} + \varepsilon_{10,i,t} . \\ \end{aligned}$$

Similarly, we replace dependent variable *RV* in Eq. (19) and conduct the mediating effect analysis on *RBV* and *Jump*, respectively.

## Empirical results

### Descriptive statistics

Table 2 presents the descriptive statistics of all variables, where the unit of *Size* is Chinese Yuan. Table 2 reveals that the average values of realized, continuous, and jump volatilities for the selected green stocks are 0.00103, 0.000767, and 0.000279, respectively. We can find that the jump volatility is relatively small compared with the *RBV*. In addition, in our sample period, the average Internet sentiment is − 0.746, revealing that investors are more inclined to post negative remarks and express pessimistic sentiment through the online social media platform. Although the mean value of trading sentiment is almost 0, its standard deviation indicates that the trading sentiment is more unstable than the Internet sentiment.

**Table 2:** Descriptive statistics

Variable	Mean	SD	Min	P25	Median	P75	Max
*RV*	0.00103	0.00339	0	0.000294	0.000509	0.000995	0.407
*RBV*	0.000767	0.00120	0	0.000234	0.000420	0.000826	0.0385
*Jump*	0.000279	0.00299	0	1.38e−05	6.06e−05	0.000154	0.389
*SentiIntern*	− 0.746	0.540	− 3.555	− 1.099	− 0.747	− 0.405	2.079
*SentiTrade*	1.42e−10	0.749	− 5.701	− 0.357	− 0.0805	0.220	8.297
*VPIN*	0.273	0.129	0	0.180	0.241	0.336	0.970
*BM*	0.583	0.338	0.0555	0.319	0.520	0.792	1.978
*Size*	2.360e+10	4.510e+10	1.590e+09	5.380e+09	9.410e+09	2.380e+10	8.180e+11
*SenNum*	63.41	103.0	0	21	38	71	4199
*Return*	5.050e−04	0.0284	− 0.223	− 0.0139	0	0.0131	0.182
*CS*	0.775	0.109	0.474	0.697	0.752	0.852	1.115
*TS*	0.680	0.259	0.314	0.518	0.582	0.770	1.470

We then conduct the data preprocessing procedure as follows. First, *RV*, *RBV,* and *Jump* are multiplied by 10^4^ for convenience. To overcome the possible problem when *RV, RBV,* and *Jump* are close to 0, we follow Huang (2018)’s volatility transformation method. Specifically, we modify the dependent variable *Y* as log(1 + *Y*), where $Y \in \{ RV,RBV,Jump\}$. The same treatment is conducted for *SenNum*, and the logarithm is taken for the variable *Size*.

We also present the correlation analysis among all variables, and the results are shown in Table 3. The correlations between the trading sentiment and price fluctuations, including realized, continuous, and jump volatilities, are higher than those of the Internet sentiment. The correlation coefficient between the Internet sentiment and jump volatility is insignificant from 0. In addition, the variable VPIN presents significantly positive correlations both with investor sentiments and with volatilities. However, the correlation coefficients between different control variables are relatively small, so it can be concluded that the possible collinearity problem is faint.

**Table 3:** Correlation analysis among variables

	*RV*	*RBV*	*Jump*	*SentiTrade*	*SentiIntern*	*VPIN*	*BM*	*Size*	*SenNum*	*Return*	*CS*	*TS*
*RV*	1											
*RBV*	0.901***	1										
*Jump*	0.651***	0.346***	1									
*SentiTrade*	0.415***	0.430***	0.203***	1								
*SentiIntern*	0.061***	0.071***	0.008	0.072***	1							
*VPIN*	0.630***	0.632***	0.320***	0.397***	0.080***	1						
*BM*	− 0.255***	− 0.290***	− 0.047***	− 0.228***	− 0.090***	− 0.193***	1					
*Size*	0.015***	0.051***	− 0.073***	− 0.002	0.076***	0.087***	− 0.267***	1				
*SenNum*	0.416***	0.409***	0.232***	0.281***	− 0.169***	0.389***	− 0.180***	0.313***	1			
*Return*	0.114***	0.116***	0.095***	0.081***	0.217***	0.165***	− 0.040***	0.042***	0.017***	1		
*CS*	0.184***	0.181***	0.091***	0.105***	− 0.057***	0.187***	− 0.006	0.011**	0.091***	− 0.005	1	
*TS*	0.071***	0.067***	0.047***	0.053***	− 0.110***	0.056***	0.054***	− 0.021***	0.060***	0.013**	0.391***	1

Next, we conduct the unit root tests, and the results are shown in Table 4. The unit root test indicates that all variables are stationary at the 1% significance level.

**Table 4:** The unit root testing results

Variable	IPS	ADF
*RV*	− 77.0527***	316.4441***
*RBV*	− 81.074***	339.7782***
*Jump*	− 130.00***	360.7965***
*SentiIntern*	− 120.00***	360.7965***
*SentiTrade*	− 74.8503***	295.7380***
*VPIN*	− 86.5357***	318.6713***
*BM*	− 3.3807***	3.5833***
*Size*	− 2.7788***	2.4525***
*SenNum*	− 71.2984***	248.2016***
*Return*	− 160.0***	360.7965***
*CS*	− 14.9998***	18.2447***
*TS*	− 120.00***	360.7965***

### Baseline regression results

To examine the impacts of investor sentiments on stock volatilities, we first estimate the parameters in Eqs. (10) to (15). The Hausman tests suggest a fixed-effect panel model, and the results of the fixed-effect regressions are shown in Table 5.

**Table 5:** Results of the impact of investor sentiment on stock volatility

	(1)	(2)	(3)	(4)	(5)	(6)
	*RV*	*RV*	*RBV*	*RBV*	*Jump*	*Jump*
*SentiIntern*		0.0447*** (6.03)		0.0582*** (7.46)		0.0398*** (4.51)
*SentiTrade*	0.0777*** (4.95)	0.0740*** (4.91)	0.100*** (5.07)	0.0952*** (5.03)	0.0809*** (4.89)	0.0755*** (4.81)
*L.Y*	0.529*** (50.19)	0.521*** (49.58)	0.421*** (33.14)	0.410*** (31.69)	0.188*** (18.33)	0.184*** (18.13)
*BM*	− 0.283*** (− 3.32)	− 0.267*** (− 3.14)	− 0.320*** (− 3.24)	− 0.296*** (− 3.01)	− 0.108* (− 1.66)	− 0.0879 (− 1.37)
*Size*	0.123*** (2.86)	0.119*** (2.80)	0.181*** (3.87)	0.177*** (3.84)	− 0.0631* (− 1.82)	− 0.0693** (− 2.04)
*SenNum*	0.0672*** (8.17)	0.0831*** (8.98)	0.105*** (9.59)	0.127*** (10.13)	0.0933*** (10.36)	0.109*** (11.39)
*Return*	0.352** (2.28)	0.196 (1.26)	0.670*** (4.50)	0.482***	0.297	0.126
*CS*	0.736*** (21.23)	0.749*** (21.74)	0.695*** (17.20)	0.711*** (17.67)	0.599*** (17.42)	0.605*** (17.49)
*TS*	− 1.471*** (− 17.91)	− 1.473*** (− 17.62)	− 1.362*** (− 16.99)	− 1.354*** (− 16.59)	− 1.485*** (− 13.17)	− 1.482*** (− 13.01)
*_cons*	− 2.621** (− 2.57)	− 2.561** (− 2.55)	− 3.897*** (− 3.52)	− 3.849*** (− 3.53)	1.256 (1.53)	1.356* (1.68)
*Chisq*	1848.80***	1863.39***	2999.80***	3079.48***	2103.07***	2118.87***
*Firm*	Control	Control	Control	Control	Control	Control
*Weekday*	Control	Control	Control	Control	Control	Control
*adj. R*^*2*^	0.418	0.419	0.359	0.361	0.085	0.086

*, **, ***Denote the 10%, 5% and 1% significance level, respectively. Firm and Weekday represent the individual and weekday effects, and *Chisq* indicates the statistics of the Hausman test. The Hausman test suggests a fixed− effect panel model at the 1% significance level

Columns (1) and (3) of Table 5 demonstrate that trading sentiment significantly increases both realized and continuous volatilities. This phenomenon reveals that when investor sentiment is high, the irrational behavior of noise traders leads to a mismatch between risk and return. Owing to the existence of short-selling restrictions, when the asset prices are overvalued, the rational arbitrageurs tend to withdraw from the overvalued trading market rather than adjust the overvalued prices. However, irrational traders may continue to execute buyer-side trades, causing asset prices to deviate further from their fundamental values. Therefore, the imbalance between supply and demand would intensify the fluctuations of stock prices, which leads to increased volatilities. After incorporating the Internet sentiment with the trading sentiment, columns (2) and (4) also reveal a significant positive relationship between Internet sentiment and realized (continuous) volatility. However, the partial effect of Internet sentiment on volatility is weaker than that of trading sentiment. This may be attributed to the limited users of the Eastmoney Guba, although it is the largest social media platform for investors. Consequently, the Internet sentiment does not affect investors who ignore this forum.

Interestingly, with the inclusion of the Internet sentiment in the models, columns (2) and (4) of Table 3 indicate that the impact of trading sentiment on the realized (continuous) volatility decreases. The Internet text discloses more information about the green stocks, which may improve the effectiveness of the green stock market and thus alleviate the impact of trading sentiment on volatilities. However, negative news conveyed in the Internet sentiment will also spread in real-time through the social media network, thus encouraging investors to buy or sell stocks. Although the Internet sentiment reduces the impact of trading sentiment on stock market volatility, its impact on green stock volatility cannot be ignored.

We also find that the jump volatility is sensitive to changes in trading and Internet sentiments in the green stock market. Specifically, price jumps are usually due to the impact of innovation information, resulting in large or even violent volatility in the short term. Sudden information shocks often cause these jumps; therefore, jump volatility contains ample information content. In addition, consistent with the realized and continuous volatilities, jump volatility is also more susceptible to trading sentiment, and the introduction of Internet sentiment decreases the impact of trading sentiment on jump volatility. The results of the effects of investor sentiment on realized, continuous, and jump volatilities are consistent with the findings of Gong et al. (2022) and Liu et al. (2022). Specifically, Gong et al. (2022) revealed that investor sentiment can significantly increase the realized volatility of stocks based on in-sample, sub-sample, and out-of-sample analysis. Liu et al. (2022) also found that Internet sentiment can significantly exacerbate price jumps. Because retail investors act as the main traders in China’s stock market, investors tend to overreact to information. When the facts are inconsistent with expectations, investors are prone to overcorrection, resulting in short-term stock price fluctuations. Moreover, the short subject of options and futures in China’s stock market is limited, the short selling mechanism is challenging to work, and stock market arbitrage is severely restricted. Irrational investors, who are triggered by the stock deviation from the fundamental phenomenon, are difficult to correct in time and can easily cause continuous or even jump volatility.

Furthermore, by analyzing the impacts of trading sentiment on continuous and jump volatility, we can conclude that trading sentiment imposes similar effects on these two volatilities. Under the current situation of an incomplete green stock market policy and credit system framework, trading sentiment can more easily amplify stock market volatility, and even cause jumps in green stock prices. As to the Internet sentiment’s effects on continuous and jump volatility, for green stocks, Internet sentiment seems more likely to trigger continuous volatility. However, the influence of Internet sentiment on jump volatility cannot be ignored. The reason for this may be that the Internet sentiment could provide investors more distinct positive or negative news, and these tend to form a consistent emotional tendency due to the silent spiral effect. This will result in a more powerful impact on the stock market, and even drive the stock price to jump in turn. As far as the green stock market is concerned, both trading and Internet sentiment can significantly increase jump volatility. The operational stability of the green stock market still needs to be improved. In summary, the above analysis verifies H_1_.

To further investigate whether there exist significant differences in investor sentiment on the stock volatilities between different stock boards in China’s market, we divide the whole sample into the Main board, SME board, and GEM board. Specifically, the Main, SME, and GEM boards include 52, 31, and 23 green stocks, respectively. The estimation results are shown in Table 6. Consistent with the conclusion for the whole sample, trading sentiment displays significant positive impacts on realized, continuous, and jump volatilities. Internet sentiment also significantly exacerbates the realized and continuous volatilities in different boards, but its impacts are weaker than those of trading sentiment. In contrast, the impact of Internet sentiment on jump volatility is only significant in the Main board, and it is insignificant in the other two boards, which may be due to the much lower number of green stocks in the SME and GEM boards.

**Table 6:** The results of investor sentiments’ effects on volatilities in different boards

	(1)	(2)	(3)	(4)	(5)	(6)
	*RV*	*RV*	*RBV*	*RBV*	*Jump*	*Jump*
*Panel A: Main board*						
*SentiIntern*		0.0437*** (4.24)		0.0486*** (4.43)		0.0450*** (4.44)
*SentiTrade*	0.0736*** (2.95)	0.0704*** (2.94)	0.0945*** (3.06)	0.0913*** (3.06)	0.0760** (2.67)	0.0704** (2.64)
*L.Y*	0.537*** (37.22)	0.531*** (37.18)	0.430*** (22.56)	0.422*** (21.88)	0.183*** (11.65)	0.178*** (11.54)
*BM*	− 0.324*** (− 2.87)	− 0.308*** (− 2.76)	− 0.330** (− 2.34)	− 0.308** (− 2.21)	− 0.205*** (− 3.09)	− 0.181*** (− 2.70)
*Size*	0.0661 (0.92)	0.0626 (0.91)	0.136 (1.57)	0.131 (1.59)	− 0.139*** (− 2.96)	− 0.146*** (− 3.27)
*SenNum*	0.0608*** (5.03)	0.0745*** (5.42)	0.100*** (6.08)	0.119*** (6.24)	0.0932*** (6.65)	0.111*** (7.43)
*Return*	0.504** (2.24)	0.343 (1.46)	0.568** (2.44)	0.416* (1.70)	0.624* (1.86)	0.432 (1.34)
*CS*	0.795*** (20.56)	0.809*** (20.68)	0.762*** (17.43)	0.778*** (17.54)	0.617*** (12.75)	0.625*** (12.74)
*TS*	− 1.470*** (− 12.42)	− 1.467*** (− 12.27)	− 1.442*** (− 12.07)	− 1.428*** (− 11.86)	− 1.307*** (− 9.24)	− 1.298*** (− 9.13)
*_cons*	− 1.349 (− 0.78)	− 1.295 (− 0.79)	− 2.956 (− 1.41)	− 2.892 (− 1.45)	3.095*** (2.75)	3.221*** (2.99)
*Firm*	Control	Control	Control	Control	Control	Control
*Weekday*	Control	Control	Control	Control	Control	Control
*adj. R*^*2*^	0.418	0.419	0.355	0.356	0.085	0.086
Panel B: SME board						
*SentiIntern*		0.0329** (2.11)		0.0614*** (3.87)		0.0321 (1.45)
*SentiTrade*	0.0834*** (2.91)	0.0803*** (2.82)	0.123*** (3.44)	0.116*** (3.28)	0.0994*** (3.96)	0.0938*** (3.75)
*L.Y*	0.541*** (25.96)	0.535*** (25.45)	0.411*** (16.17)	0.402*** (15.32)	0.214*** (10.61)	0.212*** (10.62)
*BM*	− 0.0979 (− 0.79)	− 0.0858 (− 0.69)	− 0.168 (− 1.14)	− 0.148 (− 1.00)	0.114 (1.03)	0.126 (1.13)
*Size*	0.124* (1.92)	0.122* (1.86)	0.166** (2.19)	0.165** (2.14)	− 0.0493 (− 0.82)	− 0.0529 (− 0.89)
*SenNum*	0.0706*** (5.25)	0.0834*** (5.21)	0.108*** (5.99)	0.130*** (5.92)	0.103*** (6.87)	0.117*** (7.02)
*Return*	0.406* (1.77)	0.290 (1.24)	0.874*** (4.09)	0.662*** (3.26)	0.139 (0.33)	0.0102 (0.02)
*CS*	0.784*** (10.71)	0.788*** (10.92)	0.776*** (8.66)	0.782*** (8.84)	0.610*** (8.23)	0.607*** (8.11)
*TS*	− 1.543*** (− 11.14)	− 1.551*** (− 11.01)	− 1.338*** (− 9.15)	− 1.343*** (− 8.94)	− 1.776*** (− 7.39)	− 1.776*** (− 7.38)
*_cons*	− 2.759* (− 1.85)	− 2.729* (− 1.82)	− 3.614** (− 2.08)	− 3.632** (− 2.06)	0.753 (0.53)	0.804 (0.58)
*Firm*	Control	Control	Control	Control	Control	Control
*Weekday*	Control	Control	Control	Control	Control	Control
*adj. R*^*2*^	0.442	0.443	0.369	0.371	0.104	0.105
*Panel C: GEM board*						
*SentiIntern*		0.0548*** (4.15)		0.0704*** (5.12)		0.0296 (1.65)
*SentiTrade*	0.0830*** (3.19)	0.0778*** (3.22)	0.0947*** (3.15)	0.0882*** (3.18)	0.0767*** (3.31)	0.0724*** (3.27)
*L.Y*	0.481*** (21.55)	0.469*** (21.86)	0.396*** (16.03)	0.378*** (15.11)	0.161*** (9.92)	0.157*** (9.63)
*BM*	− 0.869*** (− 3.37)	− 0.859*** (− 3.40)	− 0.996*** (− 3.79)	− 0.986*** (− 3.80)	− 0.301 (− 1.26)	− 0.280 (− 1.20)
*Size*	0.125** (2.28)	0.114** (2.20)	0.168** (2.68)	0.157** (2.63)	− 0.00748 (− 0.16)	− 0.0147 (− 0.32)
*SenNum*	0.0780*** (4.32)	0.103*** (6.96)	0.113*** (5.10)	0.146*** (6.92)	0.0736*** (4.64)	0.0877*** (5.96)
*Return*	0.106 (0.29)	− 0.0632 (− 0.18)	0.557 (1.64)	0.345 (1.05)	0.0464 (0.09)	− 0.100 (− 0.20)
*CS*	0.521*** (6.85)	0.545*** (7.15)	0.433*** (5.09)	0.461*** (5.38)	0.517*** (8.40)	0.529*** (8.98)
*TS*	− 1.394*** (− 7.03)	− 1.392*** (− 6.88)	− 1.228*** (− 7.11)	− 1.212*** (− 6.95)	− 1.530*** (− 6.23)	− 1.533*** (− 6.10)
*_cons*	− 2.166 (− 1.64)	− 1.977 (− 1.57)	− 3.057* (− 2.03)	− 2.853* (− 1.99)	0.178 (0.16)	0.299 (0.27)
*Firm*	Control	Control	Control	Control	Control	Control
*Weekday*	Control	Control	Control	Control	Control	Control
*adj. R*^*2*^	0.393	0.394	0.361	0.363	0.065	0.066

*, **, ***Denote the 10%, 5% and 1% significance level, respectively. *Firm* and *Weekday* represent the individual and time effects

### Estimation results of mediating effect models

To explore the mechanism of investor sentiments on stock volatilities more intuitively and precisely, we conduct a stepwise regression to determine the role of information asymmetry. Referring to Baron and Kenny (1986), the stepwise method is divided into three steps. Consider the realized volatility, for instance. First, we examine whether the investor sentiment is significantly related to realized volatility. The coefficient $\beta_{11}$ of Eq. (10) reflects the total effect of investor sentiment on RV. The second step is to investigate the impact of investor sentiment on information asymmetry. Finally, we explore whether investor sentiment and information asymmetry have considerable effects on realized volatility. The product of the two coefficients, $\xi_{11}$ and $\theta_{1}$, respectively, in Eqs. (16) and (17) reflect the indirect effect of investor sentiment on realized volatility, and the coefficient $\beta_{14}$ in Eq. (17) represents the direct effect of investor sentiment on realized volatility. In addition, the size of the mediating effect is yielded by $\left( {\xi_{11} \times \theta_{1} } \right)/\beta_{11}$. Columns (1) and (2), (3) and (4), and (5) and (6) of Table 5 present the first-step results of RV, RBV, and Jump, respectively. The fixed-effect estimation method is also adopted for the subsequent analysis. The results of the second step are shown in columns (1) and (5) of Table 7. Columns (2) to (4) display the RV, RBV, and Jump results for the third step in the absence of Internet sentiment, respectively. Columns (6) to (8) show the corresponding results for including both the Internet sentiment and the trading sentiment.

**Table 7:** Mediation effect model results

	(1)	(2)	(3)	(4)	(5)	(6)	(7)	(8)
	*VPIN*	*RV*	*RBV*	*Jump*	*VPIN*	*RV*	*RBV*	*Jump*
*SentiIntern*					0.00526*** (5.26)	0.0391*** (5.47)	0.0474*** (6.48)	0.0280*** (3.27)
*SentiTrade*	0.0120*** (5.58)	0.0673*** (4.72)	0.0825*** (4.76)	0.0579*** (4.23)	0.0115*** (5.62)	0.0642*** (4.68)	0.0788*** (4.72)	0.0547*** (4.17)
*VPIN*		0.746*** (9.04)	1.479*** (18.91)	0.759*** (9.83)		0.729*** (8.91)	1.449*** (18.45)	0.735*** (9.44)
*L.Y*	0.434*** (30.34)	0.476*** (37.94)	0.306*** (25.90)	0.170*** (16.25)	0.427*** (29.24)	0.470*** (38.02)	0.299*** (25.17)	0.168*** (16.26)
*BM*	− 0.066*** (− 4.02)	− 0.220*** (− 2.74)	− 0.212** (− 2.32)	− 0.0218 (− 0.34)	− 0.0641*** (− 3.94)	− 0.207** (− 2.57)	− 0.195** (− 2.11)	− 0.0110 (− 0.17)
*Size*	0.0467*** (5.20)	0.0749* (1.87)	0.0945** (2.32)	− 0.125*** (− 3.70)	0.0465*** (5.26)	0.0725* (1.83)	0.0930** (2.28)	− 0.128*** (− 3.81)
*SenNum*	0.0136*** (9.30)	0.0592*** (7.63)	0.0879*** (8.87)	0.0720*** (8.45)	0.0160*** (9.55)	0.0733*** (8.41)	0.107*** (9.33)	0.0842***
								(9.01)
*Return*	0.144*** (6.49)	0.234 (1.43)	0.454*** (3.05)	0.0878 (0.35)	0.127*** (5.73)	0.0985 (0.60)	0.306** (2.06)	− 0.0355 (− 0.15)
*CS*	0.0737*** (12.51)	0.704*** (21.11)	0.627***	0.522***	0.0749*** (12.74)	0.717*** (21.59)	0.643*** (16.57)	0.529*** (15.52)
			(16.20)	(15.47)				
*TS*	− 0.073*** (− 5.81)	− 1.437*** (− 17.48)	− 1.222*** (− 15.08)	− 1.393*** (− 12.23)	− 0.0738*** (− 5.80)	− 1.440*** (− 17.21)	− 1.219*** (− 14.77)	− 1.395*** (− 12.14)
*_cons*	− 1.009*** (− 4.73)	− 1.559 (− 1.65)	− 1.992** (− 2.07)	2.599*** (3.24)	− 1.010*** (− 4.81)	− 1.529 (− 1.63)	− 1.994** (− 2.07)	2.636*** (3.31)
*Firm*	Control	Control	Control	Control	Control	Control	Control	Control
*Weekday*	Control	Control	Control	Control	Control	Control	Control	Control
*adj. R*^*2*^	0.422	0.422	0.376	0.091	0.423	0.423	0.377	0.092

*, **, ***Denote the 10%, 5% and 1% significance level, respectively. *Firm* and *Weekday* represent the individual and time effects

Column (2) of Table 7 shows that the direct effect of trading sentiment on realized volatility is 0.0673, and the total effect of trading sentiment on realized volatility is 0.0777, according to the column (1) of Table 5. This may be because trading sentiment is positively correlated with the VPIN from the results in column (1) of Table 7. The higher trading sentiment will facilitate the informed traders, and they can obtain excess returns in the trading process. Investors with an informational advantage incorporate information into the stock price during the transaction process, thereby exacerbating the volatility of green stock prices. The VPIN performs as a transmission channel in the effect of trading sentiment on the realized volatility, and the mediating effect is 0.0120 × 0.746/0.0777, namely 11.521%.

When incorporating the Internet sentiment into the mediating effect models, both the total and direct effects of trading sentiment on realized volatility decrease slightly. Meanwhile, the VPIN’s mediating effect size also reduces to 0.0115 × 0.729/0.0740, namely 11.329%. This can be attributed to the fact that investors will adjust their investment decisions after exchanging information through social network platforms. The correlation between Internet sentiment and the VPIN is also significantly positive, indicating that the VPIN performs as an important way for Internet sentiment to affect realized volatility. This result further illustrates that the information in the stock market shows non-homogeneity due to the differences in information acquisition and processing by individual investors. The arrival of information will change investors’ expectations of assets, and the rendering of investor sentiment provides convenience for informed traders to trade, thus exacerbating the stock market’s volatility.

Similarly, we adopt the stepwise method to analyze the mediating effects of VPIN on continuous and jump volatilities, respectively. Table 6 shows that VPIN presents a mediating effect of 17.748% on the continuous volatility affected by trading sentiment. The mediating effect on jump volatility reaches 11.258%. This indicates that the VPIN, as a transmission channel for investor sentiment to affect price volatility, also plays a significant role in continuous and jump volatilities. In particular, the Chinese green stock market is still in its infancy, and the lack of financial products will cause more price fluctuations. Then maintaining good information disclosure and transparency of green stocks is of great significance for the stability of the green stock market and the alleviation of the jump occurrence. Moreover, with the inclusion of the Internet sentiment, the mediating effects of the VPIN in the trading sentiment’s influence on the continuous and jump volatilities reach 17.504% and 11.195%, respectively. Consistent with the results on the realized volatility, the introduction of Internet sentiment reduces the VPIN’s mediating effect. Besides, comparing the mediating effect of the VPIN in the role of trading sentiment’s influence on continuous volatility with that on jump volatility, it can be found that trading sentiment seems more likely to cause continuous volatility through the mediating path of the VPIN.

Besides the stepwise method conducted above, we also use the Bootstrap method following Preache and Hayes (2008) to further confirm whether the VPIN’s mediating effect is significant in the influence of investor sentiments on volatilities. The testing results are shown in Table 8, revealing that the indirect effect coefficients of trading sentiment and Internet sentiment on realized volatility, continuous volatility, and jump volatility are all greater than 0 within the 95% confidence interval. The Internet sentiment can also affect volatilities through the VPIN. Consequently, the assumptions H_2_ and H_3_ of this study are both verified.

**Table 8:** Bootstrap testing results

Explained variables	Explanatory variables	*Ind_eff Coef*	*SE*	*z*	*P* >*|z|*	95% *Conf. Interval*	
*RV*	*SentiTrade*	0.0060	0.0007	8.17	0.000	0.0046	0.0075
	*SentiIntern*	0.0041	0.0005	7.70	0.012	0.0030	0.0051
	*SentiTrade* & *SentiIntern*	0.0057	0.0008	7.57	0.000	0.0042	0.0072
*RBV*	*SentiTrade*	0.0113	0.0009	12.32	0.000	0.0095	0.0131
	*SentiIntern*	0.0074	0.0008	9.01	0.000	0.0058	0.0090
	*SentiTrade* & *SentiIntern*	0.0107	0.0009	11.93	0.000	0.0090	0.0125
*Jump*	*SentiTrade*	0.0170	0.0017	9.96	0.000	0.0136	0.0203
	*SentiIntern*	0.0115	0.0011	10.02	0.000	0.0092	0.0137
	*SentiTrade* & *SentiIntern*	0.0155	0.0016	9.65	0.000	0.0123	0.0186

*Ind_eff Coef* indicates the indirect effect point estimate, *SE* is the standard error, *z* represents the *Z* statistic, and 95% *Conf. Interval* denotes the 95% confidence interval of the point estimate

## Further analysis

The outbreak of the COVID-19 at the ending of 2019 has seriously impaired the world’s economy, and investor sentiment has become more complicated and volatile. Therefore, we divide the whole sample into pre- and post-pandemic subsamples and clarify whether there are significant differences in investor sentiment’s effects on volatilities. Since the first case was notified by the official on December 12, 2019, we selected this day as a node to divide the whole sample into before and after COVID-19 groups.

### Main regression analysis around COVID-19

First, we analyze the impact of investor sentiment on these three types of volatilities before and after the pandemic. The results are listed in Table 9.

**Table 9:** Results of baseline model before and after the COVID-19 pandemic

	(1)	(2)	(3)	(4)	(5)	(6)
	*RV*	*RV*	*RBV*	*RBV*	*Jump*	*Jump*
*Panel A: Before the COVID-19*						
*SentiIntern*		0.0284*** (2.64)		0.0287*** (2.64)		0.0274** (2.49)
*SentiTrade*	0.0955*** (4.28)	0.0925*** (4.22)	0.103*** (3.95)	0.101*** (3.86)	0.0772*** (4.10)	0.0713*** (3.99)
*L.Y*	0.369*** (20.12)	0.362*** (19.99)	0.310*** (17.23)	0.304*** (16.35)	0.156*** (7.98)	0.152*** (7.99)
*BM*	− 0.882*** (− 3.23)	− 0.899*** (− 3.43)	− 0.960*** (− 3.48)	− 0.964*** (− 3.59)	− 0.237 (− 0.99)	− 0.242 (− 1.03)
*Size*	0.0284 (0.22)	0.00456 (0.04)	0.136 (0.89)	0.116 (0.79)	− 0.145* (− 1.75)	− 0.168** (− 2.12)
*SenNum*	0.0494*** (4.15)	0.0665*** (4.96)	0.0656*** (4.97)	0.0828*** (5.46)	0.0527*** (4.61)	0.0704*** (5.66)
*Return*	1.808*** (6.58)	1.727*** (6.53)	1.984*** (6.82)	1.931*** (6.64)	0.967** (2.32)	0.858** (2.13)
*CS*	0.363*** (2.91)	0.381*** (3.11)	0.381*** (3.36)	0.407*** (3.62)	0.255* (1.72)	0.257* (1.72)
*TS*	− 0.549** (− 2.61)	− 0.557*** (− 2.65)	− 0.827*** (− 4.06)	− 0.820*** (− 4.03)	− 0.157 (-0.64)	− 0.163 (-0.66)
*_cons*	0.476 (0.15)	0.996 (0.33)	− 2.046 (− 0.55)	− 1.612 (− 0.46)	3.633* (1.75)	4.120** (2.07)
*Firm*	Control	Control	Control	Control	Control	Control
*Weekday*	Control	Control	Control	Control	Control	Control
*adj. R*^*2*^	0.234	0.235	0.212	0.215	0.050	0.051
*Panel B: After the COVID-19*						
*SentiIntern*		0.0508*** (5.66)		0.0722*** (7.92)		0.0417*** (3.55)
*SentiTrade*	0.0824*** (3.92)	0.0783*** (3.94)	0.113*** (3.96)	0.107*** (4.01)	0.0965*** (4.39)	0.0913*** (4.37)
*L.Y*	0.543*** (45.89)	0.535*** (46.20)	0.406*** (24.88)	0.393*** (24.66)	0.183*** (16.32)	0.179*** (16.00)
*BM*	− 0.406*** (− 6.20)	− 0.386*** (− 5.99)	− 0.435*** (− 5.47)	− 0.405*** (− 5.10)	− 0.258*** (− 3.89)	− 0.234*** (− 3.53)
*Size*	0.0668 (1.65)	0.0602 (1.51)	0.139*** (2.76)	0.129** (2.61)	− 0.188*** (− 4.93)	− 0.196*** (− 5.17)
*SenNum*	0.0688*** (7.16)	0.0848*** (8.32)	0.126*** (9.08)	0.153*** (10.15)	0.108*** (9.23)	0.123*** (10.06)
*Return*	− 0.0661 (− 0.40)	− 0.246 (− 1.47)	0.191 (1.20)	− 0.0443 (− 0.28)	0.122 (0.48)	− 0.0568 (− 0.22)
*CS*	0.636*** (16.12)	0.644*** (16.52)	0.571*** (11.95)	0.581*** (12.32)	0.578*** (13.93)	0.581*** (14.10)
*TS*	− 1.564*** (− 18.07)	− 1.558*** (− 17.74)	− 1.323*** (− 15.54)	− 1.304*** (− 15.05)	− 1.723*** (− 14.38)	− 1.716*** (− 14.22)
*_cons*	− 1.164 (− 1.21)	− 1.033 (− 1.10)	− 2.756** (− 2.31)	− 2.577** (− 2.20)	4.215*** (4.64)	4.356*** (4.85)
*Firm*	Control	Control	Control	Control	Control	Control
*Weekday*	Control	Control	Control	Control	Control	Control
*adj. R*^*2*^	0.415	0.416	0.332	0.335	0.087	0.088

*, **, ***Denote the 10%, 5% and 1% significance level, respectively. *Firm* and *Weekday* represent the individual and time effects

According to the results before and after the COVID-19 in Table 9, we confirm that investor sentiment positively affects realized volatility in the two subsamples. That is, higher investor sentiment produces more volatile green stock markets, which is consistent with the conclusion in Sect. 4. However, by comparing the coefficients before and after the COVID-19, we can find that investor sentiment has a severer impact on volatilities after the outbreak of COVID-19 in general. Specifically, the results reveal that the coefficient of Internet sentiment on realized volatility before the pandemic is 0.0284, while the coefficient after the COVID-19 reaches 0.0508, increasing to 1.789 times that before the pandemic. The reasons for this phenomenon may lie in two aspects. On the one hand, dual carbon targets have not yet been proposed before the outbreak of COVID-19, and green stocks attracted less attention with fewer posts in Eastmoney Guba. This can also be reflected in the volume of the posts. The number of posts also imposes insignificant impacts on volatility before the pandemic. On the other hand, the outbreak was sudden. Investors knew little about the virus and were hungry for information. At this time, gossip and rumors spread more easily, and pessimism and panic made investors more likely to sell stocks, resulting in intense stock price fluctuations. Consequently, stock price fluctuations after the pandemic are more susceptible to the Internet sentiment. Moreover, affected by the pandemic, the panic generated on the Internet spreads rapidly after being brewed, causing a sudden impact on stock price fluctuations. Financial asset volatilities tend to display leverage effects. Bad news usually brings more intense volatility than good news does, so the Internet sentiment after the pandemic is more likely to generate price volatility.

Similarly, to verify the robustness of our empirical results, we further analyze the difference in the impact of investor sentiment on continuous and jump volatilities around the COVID-19. The corresponding results are shown in columns (3–6) of Table 9. By analyzing Panels A and B in Table 9, We find that the impacts of Internet sentiment on continuous and jump volatilities increased significantly, which reach 2.516 and 1.522 times that before the pandemic, respectively. The influence of trading sentiment on continuous and jump volatilities has also increased slightly after the epidemic. The impact of COVID-19 on stock market volatility is also described in the relevant literature. For example, John and Li (2021) analyze the impact of different types of news on the jump component in the VIX index and the jump component in realized volatility, and the results showed that COVID-19 and the market’s Google search index increased the jump in the VIX index and realized volatility. Liu et al. (2022) also confirm that extreme Internet sentiment is more prone to jump. Moreover, continuous volatility is more sensitive to investor sentiment than jump volatility, which further verifies the results of Sect. 4.2.

### Mediating effect

The results in Sect. 4.2 show that information asymmetry can enhance the impact of investor sentiment on volatility. We further analyze the differences in the mediating role of VPIN on the path of investor sentiment affecting volatilities around the COVID-19. Section 5.1 has presented the results of the first step in the mediating effect analysis. Specifically, columns (1) and (2), (3) and (4), and (5) and (6) in Table 9, display the total effect results on realized, continuous, and jump volatilities, respectively. Fixed-effect estimation is conducted in the subsequent analysis. Table 10 shows the mediating effect results, among which columns (1) and (5) of Table 10 are the results of the second step. Columns (2) to (4) are the third-step mediation results of RV, RBV, and Jump without Internet sentiment, respectively. Columns (6) to (8) show the corresponding results with the inclusion of Internet sentiment.

**Table 10:** Results of mediation effect model before and after the COVID-19 pandemic

	(1)	(2)	(3)	(4)	(5)	(6)	(7)	(8)
	*VPIN*	*RV*	*RBV*	*Jump*	*VPIN*	*RV*	*RBV*	*Jump*
*Panel A: Mediating effect results before COVID-19*								
*SentiIntern*					0.00340*** (2.99)	0.0249** (2.33)	0.0239** (2.27)	0.0233** (2.11)
*SentiTrade*	0.0115*** (4.98)	0.0855*** (4.17)	0.0876*** (3.78)	0.0650*** (4.00)	0.0111*** (4.88)	0.0828*** (4.13)	0.0864*** (3.71)	0.0602*** (3.97)
*VPIN*		0.693*** (5.58)	1.192*** (9.31)	0.451*** (3.15)		0.691*** (5.64)	1.158*** (9.09)	0.431*** (3.05)
*L.Y*	0.417*** (24.30)	0.324*** (16.69)	0.224*** (11.92)	0.145*** (7.23)	0.409*** (23.60)	0.318*** (17.50)	0.221*** (11.61)	0.142*** (7.35)
*BM*	− 0.132*** (− 3.29)	− 0.772*** (− 3.08)	− 0.790*** (− 3.43)	− 0.137 (− 0.59)	− 0.134*** (− 3.44)	− 0.790*** (− 3.25)	− 0.800*** (− 3.55)	− 0.148 (− 0.64)
*Size*	0.0452 (1.64)	− 0.0250 (− 0.22)	0.0507 (0.42)	− 0.187** (− 2.42)	0.0430 (1.60)	− 0.0469 (− 0.43)	0.0354 (0.30)	− 0.205*** (− 2.74)
*SenNum*	0.00780*** (5.08)	0.0430*** (3.85)	0.0534*** (4.51)	0.0436*** (4.14)	0.0101*** (5.82)	0.0582*** (4.59)	0.0679*** (4.95)	0.0594*** (5.04)
*Return*	0.154*** (4.37)	1.727*** (5.99)	1.659*** (5.61)	0.875** (2.09)	0.148*** (4.20)	1.655*** (5.99)	1.632*** (5.53)	0.783* (1.94)
*CS*	0.0172 (1.30)	0.360*** (2.93)	0.381*** (3.47)	0.235 (1.62)	0.0180 (1.35)	0.376*** (3.11)	0.404*** (3.70)	0.235 (1.61)
*TS*	− 0.0250 (− 0.98)	− 0.558*** (− 2.67)	− 0.828*** (− 4.11)	− 0.144 (− 0.58)	− 0.0262 (− 1.03)	− 0.569*** (− 2.73)	− 0.825*** (− 4.10)	− 0.153 (− 0.62)
*_cons*	− 0.882 (− 1.34)	1.604 (0.57)	− 0.240 (− 0.08)	4.484** (2.32)	− 0.834 (− 1.30)	2.084 (0.79)	0.0819 (0.03)	4.886** (2.61)
*Firm*	Control	Control	Control	Control	Control	Control	Control	Control
*Weekday*	Control	Control	Control	Control	Control	Control	Control	Control
*adj. R*^*2*^	0.326	0.238	0.224	0.052	0.328	0.239	0.226	0.052
*Panel B: Mediating effect results after COVID-19*								
*SentiIntern*					0.00609*** (5.05)	0.0459*** (5.27)	0.0603*** (6.99)	0.0289** (2.55)
*SentiTrade*	0.0139*** (3.94)	0.0736*** (3.86)	0.0956*** (3.91)	0.0721*** (4.03)	0.0132*** (3.96)	0.0702*** (3.88)	0.0905*** (3.96)	0.0693*** (4.04)
*VPIN*		0.602*** (6.11)	1.413*** (14.79)	0.750*** (7.65)		0.580*** (5.92)	1.381*** (14.52)	0.723*** (7.30)
*L.Y*	0.386*** (21.80)	0.501*** (35.64)	0.298*** (21.65)	0.167*** (14.35)	0.378*** (21.49)	0.495*** (35.55)	0.289*** (21.32)	0.165*** (14.17)
*BM*	− 0.0779*** (− 4.44)	− 0.361*** (− 6.09)	− 0.346*** (− 4.60)	− 0.176** (− 2.62)	− 0.0753*** (− 4.30)	− 0.344*** (− 5.83)	− 0.323*** (− 4.24)	− 0.161** (− 2.38)
*Size*	0.0510*** (5.09)	0.0204 (0.53)	0.0421 (0.93)	− 0.256*** (− 6.58)	0.0506*** (5.08)	0.0162 (0.43)	0.0362 (0.80)	− 0.259*** (− 6.66)
*SenNum*	0.0151*** (7.94)	0.0634*** (6.88)	0.112*** (8.68)	0.087*** (7.66)	0.0175*** (8.64)	0.0781*** (8.02)	0.136*** (9.70)	0.098*** (8.08)
*Return*	0.136*** (5.56)	− 0.160 (− 0.92)	0.0581 (0.37)	− 0.0723 (− 0.27)	0.115*** (4.71)	− 0.321* (− 1.82)	− 0.135 (− 0.88)	− 0.200 (− 0.74)
*CS*	0.0588*** (8.74)	0.618*** (16.44)	0.522*** (11.58)	0.521*** (13.20)	0.0594*** (8.94)	0.626*** (16.81)	0.531*** (11.90)	0.526*** (13.22)
*TS*	− 0.0674*** (− 4.81)	− 1.537*** (− 17.91)	− 1.184*** (− 13.87)	− 1.638*** (− 13.60)	− 0.0669*** (− 4.76)	− 1.532*** (− 17.58)	− 1.171*** (− 13.48)	− 1.638*** (− 13.50)
*_cons*	− 1.081*** (− 4.54)	− 0.146 (− 0.16)	− 0.627 (− 0.58)	5.695*** (6.15)	− 1.075*** (− 4.55)	− 0.0668 (− 0.08)	− 0.528 (− 0.49)	5.734*** (6.21)
*Firm*	Control	Control	Control	Control	Control	Control	Control	Control
*Weekday*	Control	Control	Control	Control	Control	Control	Control	Control
*adj. R*^*2*^	0.347	0.418	0.349	0.092	0.348	0.418	0.351	0.093

Regardless of the Internet sentiment’s role, the impact of trading sentiment on the VPIN improved after COVID-19. When the COVID-19 pandemic suddenly occurred, the investment demand of shareholders decreased, and the liquidity of the stock market turned short. Meanwhile, the high trading sentiment activated the stock market and facilitated informed traders to complete transactions. Therefore, the influence of trading sentiment on the VPIN was strengthened. Specifically, the mediating effect of the VPIN in the impact of trading sentiment on *RV* before COVID-19 was 8.345% (0.0115 × 0.693/0.0955). After COVID-19, the VPIN’s mediating effect rose to 10.155% (0.0139 × 0.602/0.0824). The VPIN’s mediating effect after COVID-19 increased in the realized volatility affected by the trading sentiment, indicating that information transparency plays as an important role in reducing volatility and preventing risks in uncertain times. By exploring the role of Internet sentiment, we can see that both the direct effect and total effect of Internet sentiment on realized volatility increased after the COVID-19 pandemic. Consistent with trading sentiment, Internet sentiment is positively correlated with the VPIN, indicating that the Internet sentiment can directly affect realized volatility and indirectly aggravate realized volatility through the transmission of VPIN.

Furthermore, the immediate and aggregate impacts of trading sentiment on both continuous and jump volatilities intensified after COVID-19. A similar conclusion can be drawn for Internet sentiment. Consistent with the results on the realized volatility, the mediating effects of VPIN on the continuous and jump volatility of trading sentiment also improved after the COVID-19 pandemic. After calculation, it is found that the VPIN plays a stronger role in the influence of trading sentiment on continuous volatility than on jump volatility.

## Conclusion

Our study constructed both Internet sentiment and trading sentiment of investors based on multi-source data. We established fixed-effect panel data models to explore the influential mechanism and path of the two investor sentiment proxies on realized, continuous, and jump volatilities, respectively. We have drawn the following four conclusions.

First, an upsurge in trading sentiment can significantly increase realized, continuous, and jump volatilities. Continuous volatility is the most sensitive to trading or Internet sentiment, and jump volatility in the green stock market is also easily affected by investor sentiment. Second, the impacts of Internet sentiment on realized, continuous, and jump volatilities have significantly increased in the post-pandemic period. Before the pandemic, the role of Internet sentiment is limited because of lower posting volume and insufficient attention to green stocks. However, the addition of Internet sentiment discloses more information, improves the efficiency of the stock market, and thus reduces the impact of trading sentiment on volatility. Third, the impacts of trading sentiment on volatilities in different stock boards are consistent, while the Internet sentiment tends to impose limited effects on the jump volatility for the SME and GEM boards. Finally, the VPIN functions as an intermediary path through which investor sentiment affects volatilities. Investor sentiment can further amplify stock volatility by aggravating the level of information asymmetry.

Developing green stocks is the first step on the inevitable path toward a structural adjustment of the economy and the realization of economically and environmentally sustainable development. However, there remain some imperfections in China’s green stock market. For example, a standard, unified definition of green projects should be created, which will help investors make better decisions. In addition, because of the lack of a perfect information disclosure and sharing mechanism, the form and content of enterprise information disclosure vary among enterprises. The quality of information disclosed also needs to be improved, which can further enhance investors’ desire to trade. Combined with the current situation and our results, we provide an essential reference for regulators to maintain the stable development of the green stock market. On the one hand, regulators should establish scientific and efficient investor sentiment measures to minimize the negative influence of irrational sentiment, such as causing prices to deviate from fundamental values too much. On the other hand, companies should pay more attention to online forums and try to improve information disclosure for green stocks. The increase in information transparency can contribute to reducing the volatility of the stock market and avoiding systemic financial risks, and a green stock market will also be helpful in attracting financial capital for environmental protection industries.

## Data Availability

We are sorry that the authors do not have the license to share the data.

## Appendix

See Table 11.

**Table 11:** The Wind ESG ratings and scores for 2020

ID	Rating	Score	ID	Rating	Score	ID	Rating	Score
000,027.SZ	BBB	6.88	002,564.SZ	BB	5.7	600,125.SH	A	7.7
000,338.SZ	A	7.67	002,610.SZ	BBB	5.9	600,143.SH	BBB	6.37
000,400.SZ	BB	5.31	002,613.SZ	BB	5.95	600,151.SH	A	7.76
000,488.SZ	BB	5.47	002,630.SZ	BBB	6.56	600,189.SH	BB	5.1
000,540.SZ	BBB	6.85	002,639.SZ	BB	5.57	600,217.SH	BB	5.73
000,591.SZ	BBB	6.25	002,642.SZ	B	4.25	600,256.SH	BBB	6.59
000,811.SZ	BB	5.9	002,645.SZ	BB	5.54	600,273.SH	BBB	6.83
000,826.SZ	BBB	6.12	002,665.SZ	BB	5.62	600,277.SH	BBB	6.67
000,862.SZ	BB	5.75	300,001.SZ	BB	5.34	600,309.SH	BBB	6.54
000,928.SZ	BBB	6.06	300,007.SZ	A	7.31	600,339.SH	BB	5.28
000,966.SZ	BBB	6.13	300,014.SZ	BBB	6.44	600,406.SH	BBB	6.07
000,990.SZ	BB	5.3	300,040.SZ	BB	5.07	600,438.SH	BBB	6.77
000,993.SZ	BB	5.88	300,070.SZ	BBB	6.79	600,481.SH	BBB	6.03
002,050.SZ	BBB	6.18	300,072.SZ	BBB	7.03	600,516.SH	BB	5.5
002,063.SZ	A	7.59	300,080.SZ	BBB	6.84	600,517.SH	BBB	6.55
002,074.SZ	BBB	6.2	300,085.SZ	BBB	6.04	600,537.SH	BBB	6.44
002,092.SZ	BBB	6.05	300,118.SZ	BBB	6.36	600,710.SH	A	6.96
002,118.SZ	BBB	6.09	300,137.SZ	BB	5.3	600,770.SH	BB	5.56
002,126.SZ	A	7.29	300,203.SZ	BB	5.16	600,871.SH	A	7.08
002,129.SZ	BBB	6.55	300,266.SZ	BB	5.29	600,875.SH	BBB	6.84
002,130.SZ	BB	5.66	300,274.SZ	BBB	6.23	600,963.SH	BB	5.73
002,169.SZ	BBB	6.17	300,316.SZ	BBB	6.57	600,989.SH	BBB	6.88
002,178.SZ	BBB	6.71	300,345.SZ	BB	5.29	601,012.SH	A	7.59
002,202.SZ	A	7.72	300,355.SZ	BB	5.51	601,016.SH	BB	5.66
002,272.SZ	BBB	6.36	300,356.SZ	BB	5.11	601,118.SH	BBB	6.33
002,298.SZ	BBB	6.61	300,365.SZ	A	7.04	601,200.SH	BBB	6.94
002,340.SZ	A	7.37	300,417.SZ	BBB	6.63	601,222.SH	BB	5.91
002,364.SZ	BB	5.53	300,450.SZ	BB	5.29	601,330.SH	AA	8.24
002,366.SZ	BB	5.64	300,457.SZ	BB	5.57	601,615.SH	BBB	6.71
002,421.SZ	BBB	6.34	300,675.SZ	BBB	6.6	601,727.SH	AA	8.53
002,459.SZ	A	6.93	300,750.SZ	A	7.89	601,908.SH	B	4.92
002,460.SZ	AA	8.12	600,039.SH	BBB	6.1	601,985.SH	BBB	6.34
002,466.SZ	A	7.5	600,076.SH	BB	5.73	603,105.SH	BBB	6.44
002,479.SZ	BBB	6.87	600,089.SH	BBB	6.97	603,686.SH	BBB	6.35
002,506.SZ	BB	5.12	600,096.SH	A	7.58	603,993.SH	A	7.86
002,554.SZ	BB	5.48						

## Abbreviations

VPIN: Volume-synchronized probability of informed trading
COVID-19: Coronavirus disease 2019
PCA: Principal component analysis
BERT: Bidirectional encoder representations from transformers
RV: Realized volatility
RBV: Realized Bi-power variation
